# Supplementary material for: A Likelihood Approach for Real-Time Calibration of Stochastic Compartmental Epidemic Models
Source: PLoS Comput Biol. 2017 Jan 17;13(1):e1005257. doi: 10.1371/journal.pcbi.1005257 (PMC5240920; doi:10.1371/journal.pcbi.1005257)
Supplement: S1 File — (TAR.GZ) [file pcbi.1005257.s014.tar.gz › HSPH_Online-SI-Revision/PF08/n1-severe/n1-severe_PF_table-8.pdf]

1	R0	{1.51081, 13.7078}	{1.81427, 13.5445}	{{1.11228, 6.55128}, {2.59907, 19.0269}}	{32.331, 25.0159}	inf	20.	77.4265	{3.85453, 182.522}	317.626	0.61832	newly	8.	39.01	{4., 81.}	406.375	0.701496	Reff	1.3839	1.26288	{0.789151, 1.94828}	31.8224	ar	656.	635.01	{219., 906.}	31.0198
2	R0	{1.73182, 11.3873}	{1.78167, 13.0719}	{{1.07662, 6.31117}, {2.73737, 19.0443}}	{29.5663, 35.5334}	inf	10.	72.7542	{6.295, 168.25}	642.523	0.738588	newly	5.	41.83	{5., 90.}	746.2	0.782108	Reff	1.66774	1.34886	{0.872175, 2.0885}	28.7782	ar	653.	603.55	{175., 905.}	34.3936
3	R0	{1.50449, 10.3474}	{1.81208, 13.0917}	{{1.05743, 6.88118}, {2.5898, 18.8228}}	{35.3437, 42.5398}	inf	24.	74.6662	{6.08098, 159.976}	246.372	0.544553	newly	17.	41.87	{3., 96.}	185.	0.51113	Reff	1.41873	1.34088	{0.843719, 1.9328}	26.9652	ar	651.	629.57	{177., 905.}	34.6989
4	R0	{1.63493, 9.1955}	{1.9275, 13.311}	{{1.12882, 6.31117}, {2.79269, 19.0269}}	{33.4974, 59.1018}	inf	6.	56.345	{4.78513, 146.392}	852.413	0.790254	newly	4.	36.17	{3., 88.}	819.25	0.796117	Reff	1.61368	1.63059	{1.0641, 2.23343}	25.6288	ar	663.	654.45	{137., 917.}	34.1342
5	R0	{1.53822, 8.05553}	{1.82403, 13.3245}	{{1.10693, 6.62607}, {2.64373, 19.0269}}	{32.9752, 73.1977}	inf	7.	59.0522	{3.69964, 150.177}	759.047	0.793526	newly	6.	34.35	{2., 75.}	492.167	0.716857	Reff	1.51515	1.50991	{0.961356, 2.17624}	26.5883	ar	621.	641.89	{236., 908.}	35.9436
6	R0	{1.69374, 17.0793}	{1.74001, 13.0461}	{{1.11461, 5.62019}, {2.67299, 18.9813}}	{26.0904, 28.2499}	inf	15.	68.545	{3.85453, 165.044}	388.163	0.669798	newly	6.	36.04	{3., 83.}	520.333	0.755864	Reff	1.64801	1.24195	{0.764875, 1.94067}	34.4662	ar	662.	593.9	{220., 907.}	32.0544
7	R0	{1.42179, 11.8031}	{1.81524, 13.456}	{{1.11461, 6.0993}, {2.77502, 19.0269}}	{38.4382, 35.0612}	inf	25.	74.4335	{3.85453, 165.044}	239.952	0.582048	newly	16.	37.4	{3., 83.}	171.25	0.536058	Reff	1.34786	1.28194	{0.77803, 1.96011}	30.6394	ar	517.	613.49	{219., 926.}	50.1257
8	R0	{1.46683, 6.95875}	{1.93623, 13.3804}	{{1.12882, 6.55128}, {2.79269, 19.0269}}	{41.6587, 97.8372}	inf	9.	57.5637	{4.78513, 146.392}	561.498	0.689016	newly	10.	36.73	{3., 89.}	299.3	0.564337	Reff	1.44043	1.63447	{1.06485, 2.23343}	29.9202	ar	653.	657.4	{137., 917.}	35.0107
9	R0	{1.34525, 11.505}	{1.76772, 13.1716}	{{1.11523, 6.0993}, {2.59907, 19.0531}}	{38.3543, 35.5814}	inf	14.	63.0992	{3.79943, 155.247}	381.572	0.669913	newly	10.	34.36	{2., 75.}	273.4	0.635598	Reff	1.2874	1.33361	{0.78291, 1.96045}	30.2731	ar	532.	600.12	{219., 908.}	45.2669
10	R0	{1.77958, 14.372}	{1.80401, 13.8392}	{{1.15689, 6.71734}, {2.52651, 19.1171}}	{23.7724, 24.0447}	inf	20.	67.4727	{3.85453, 155.247}	270.25	0.603036	newly	5.	35.19	{3., 75.}	622.2	0.833295	Reff	1.68348	1.358	{0.801654, 1.96354}	30.8183	ar	671.	628.35	{220., 906.}	29.5365
11	R0	{1.60334, 19.9548}	{1.73461, 15.1182}	{{1.22676, 8.00549}, {2.39235, 20.}}	{23.1434, 24.3176}	inf	26.	89.4597	{25.8844, 173.786}	246.771	0.445074	newly	11.	42.27	{15., 84.}	285.727	0.505593	Reff	1.48149	1.15755	{0.744167, 1.51773}	28.9087	ar	617.	767.61	{524., 1000.}	32.1702
12	R0	{1.68623, 7.58585}	{2.00426, 12.5001}	{{1.21457, 6.01426}, {2.80569, 18.9406}}	{31.6317, 74.1574}	inf	4.	51.0656	{2.57459, 120.356}	1191.81	0.959479	newly	7.	43.29	{1., 108.}	546.429	0.754687	Reff	1.67443	1.75265	{1.13169, 2.46614}	24.938	ar	640.	670.66	{69., 928.}	36.6406
13	R0	{1.82689, 18.0022}	{1.77695, 13.322}	{{1.11461, 5.62019}, {2.75598, 19.0269}}	{26.6901, 28.5113}	inf	23.	71.4498	{3.85453, 165.044}	249.884	0.595873	newly	6.	35.86	{3., 76.}	517.	0.760559	Reff	1.74285	1.26431	{0.764875, 1.96011}	35.5926	ar	673.	603.07	{220., 918.}	32.2244
14	R0	{1.75911, 9.16883}	{1.96901, 12.3389}	{{1.20105, 6.01426}, {2.78218, 18.8445}}	{28.3266, 49.4343}	inf	4.	51.1176	{2.57459, 122.239}	1193.65	0.948751	newly	3.	42.99	{1., 108.}	1352.33	0.988594	Reff	1.75031	1.71812	{1.12479, 2.43684}	23.839	ar	661.	657.97	{69., 919.}	34.8638
15	R0	{1.46168, 11.3859}	{1.67605, 13.4478}	{{1.11228, 5.62019}, {2.24606, 19.0269}}	{27.1523, 40.6685}	inf	25.	72.2969	{2.48733, 167.7}	228.58	0.581982	newly	14.	37.24	{3., 77.}	198.714	0.542695	Reff	1.32428	1.1465	{0.792505, 1.56433}	25.4447	ar	556.	600.	{245., 862.}	36.2122
16	R0	{1.53577, 11.7581}	{2.00034, 12.3357}	{{1.20105, 6.01426}, {2.80569, 18.9248}}	{38.9532, 32.0488}	inf	5.	48.5903	{2.57459, 107.392}	889.94	0.866387	newly	2.	42.19	{1., 105.}	2026.5	1.1201	Reff	1.52041	1.75778	{1.12756, 2.46614}	29.8351	ar	625.	664.57	{69., 928.}	39.224
17	R0	{1.70698, 9.72924}	{1.97348, 12.2997}	{{1.18939, 5.7296}, {2.80569, 18.9406}}	{30.998, 45.0504}	inf	8.	47.0982	{3.78379, 105.973}	511.36	0.718331	newly	5.	41.33	{3., 101.}	747.8	0.808457	Reff	1.68138	1.73554	{1.10239, 2.4542}	25.8199	ar	675.	656.1	{69., 928.}	34.3289
18	R0	{1.5973, 14.931}	{1.97502, 12.4744}	{{1.21457, 6.01426}, {2.78218, 18.9248}}	{33.8659, 27.8294}	inf	9.	50.1214	{2.57459, 120.356}	482.54	0.711943	newly	7.	42.47	{1., 108.}	535.	0.741903	Reff	1.57973	1.72756	{1.12756, 2.43684}	26.1769	ar	632.	662.82	{69., 919.}	37.3829
19	R0	{1.87468, 17.004}	{1.75263, 14.591}	{{1.23818, 8.0269}, {2.39382, 20.}}	{22.6792, 23.2435}	inf	36.	92.1229	{27.852, 197.237}	167.014	0.358191	newly	15.	46.15	{15., 81.}	212.333	0.426753	Reff	1.69659	1.22293	{0.815217, 1.68092}	31.3461	ar	692.	780.93	{534., 1013.}	25.8743
20	R0	{1.84715, 12.531}	{1.80329, 14.9349}	{{1.24195, 7.20208}, {2.39008, 20.}}	{23.6277, 38.5612}	inf	31.	94.476	{23.4377, 182.925}	214.965	0.413496	newly	22.	46.37	{13., 83.}	127.409	0.328671	Reff	1.71785	1.26556	{0.798704, 1.74605}	31.0001	ar	661.	793.82	{507., 1026.}	31.1256
21	R0	{1.75938, 13.5959}	{1.94986, 12.3203}	{{1.16434, 5.40945}, {2.80569, 19.0269}}	{29.1503, 30.204}	inf	12.	45.8162	{3.78379, 103.519}	310.864	0.602389	newly	10.	41.68	{3., 101.}	354.2	0.650921	Reff	1.71891	1.71832	{1.08384, 2.4542}	25.471	ar	620.	643.76	{69., 928.}	40.029
22	R0	{1.63456, 10.7469}	{1.62008, 13.0816}	{{1.11365, 6.05643}, {2.14082, 18.9813}}	{20.5825, 41.5383}	inf	9.	61.9301	{2.57939, 140.409}	609.685	0.752737	newly	9.	33.38	{3., 69.}	297.111	0.596422	Reff	1.57572	1.13725	{0.76354, 1.59714}	32.0766	ar	654.	575.74	{242., 831.}	29.0061
23	R0	{1.62702, 19.1392}	{1.82037, 13.4776}	{{1.07195, 7.10524}, {2.59755, 17.5057}}	{29.6144, 29.671}	inf	40.	88.4631	{6.08098, 202.343}	166.503	0.520072	newly	13.	45.98	{4., 96.}	280.769	0.577092	Reff	1.4692	1.2484	{0.813514, 1.74375}	26.2012	ar	600.	638.85	{188., 898.}	38.895
24	R0	{1.70466, 12.1202}	{1.70487, 13.4246}	{{1.11228, 6.05643}, {2.47514, 18.9813}}	{23.5132, 34.5358}	inf	32.	78.5674	{2.41434, 167.585}	185.663	0.539348	newly	23.	38.69	{3., 78.}	111.609	0.456386	Reff	1.43191	1.0292	{0.647201, 1.4299}	32.9453	ar	613.	593.7	{184., 889.}	32.2414
25	R0	{1.61735, 11.3169}	{1.62638, 14.2002}	{{1.19222, 7.41417}, {2.39088, 20.}}	{22.5535, 40.3981}	inf	25.	77.5687	{25.0058, 165.29}	219.608	0.425083	newly	15.	36.64	{12., 83.}	152.133	0.336053	Reff	1.53163	1.11291	{0.784508, 1.41156}	29.8858	ar	681.	714.83	{464., 987.}	23.6549
26	R0	{1.56977, 12.3035}	{1.81859, 13.3019}	{{1.10693, 6.62607}, {2.64373, 19.0269}}	{31.4895, 29.1742}	inf	16.	58.714	{3.69964, 150.177}	295.013	0.562341	newly	8.	34.02	{2., 75.}	348.	0.628244	Reff	1.53367	1.50458	{0.961356, 2.17624}	26.3741	ar	594.	638.79	{236., 908.}	38.766
27	R0	{1.46317, 5.8298}	{1.8001, 12.9936}	{{1.09005, 6.74711}, {2.79269, 18.834}}	{36.8736, 125.531}	inf	8.	79.3101	{5.87089, 187.755}	903.732	0.858121	newly	8.	45.27	{4., 94.}	482.875	0.685775	Reff	1.39294	1.34343	{0.872175, 2.0885}	26.672	ar	586.	619.01	{208., 914.}	39.0222
28	R0	{1.70002, 14.9806}	{1.99779, 12.4424}	{{1.21457, 6.01426}, {2.79269, 18.9248}}	{30.7513, 27.5755}	inf	4.	50.4513	{2.57459, 120.356}	1176.45	0.95007	newly	0.1	43.06	{1., 108.}	42974.	2.23082	Reff	1.68812	1.74835	{1.13169, 2.4542}	24.4898	ar	652.	669.55	{69., 924.}	35.0844
29	R0	{1.52729, 16.1014}	{1.8136, 14.6926}	{{1.24745, 8.95398}, {2.54744, 20.}}	{29.1608, 21.9568}	inf	40.	100.4	{23.741, 196.833}	168.441	0.374608	newly	22.	47.39	{14., 87.}	128.864	0.323224	Reff	1.40358	1.1598	{0.766182, 1.66539}	25.1972	ar	592.	800.43	{539., 1018.}	40.2753
30	R0	{1.72795, 13.5635}	{1.66683, 13.8408}	{{1.11228, 6.0993}, {2.24606, 19.0443}}	{22.9204, 29.5525}	inf	42.	73.1942	{2.5376, 169.729}	126.886	0.51333	newly	22.	36.66	{3., 76.}	111.818	0.471591	Reff	1.5396	1.14345	{0.794897, 1.54705}	30.046	ar	668.	590.29	{244., 870.}	28.3907
31	R0	{1.51591, 18.1544}	{1.79652, 13.3578}	{{1.11461, 6.0993}, {2.59907, 19.2972}}	{31.618, 28.853}	inf	41.	72.1654	{3.85453, 165.044}	128.78	0.515195	newly	22.	37.11	{3., 76.}	112.864	0.488332	Reff	1.4007	1.29295	{0.778167, 1.96045}	30.9249	ar	541.	620.1	{219., 906.}	44.5619
32	R0	{1.4282, 10.3534}	{1.80704, 13.2513}	{{1.11228, 6.31117}, {2.73737, 18.4042}}	{36.4907, 43.6727}	inf	4.	75.2511	{6.98202, 159.843}	1786.55	1.08248	newly	3.	43.59	{5., 94.}	1358.33	0.996984	Reff	1.40535	1.3609	{0.872175, 2.0885}	26.1515	ar	521.	618.55	{197., 905.}	46.1785
33	R0	{1.56693, 11.3972}	{1.99086, 12.4016}	{{1.20105, 6.01426}, {2.80569, 18.9406}}	{36.5381, 34.1568}	inf	11.	47.9532	{2.57459, 106.153}	364.318	0.638313	newly	6.	41.86	{1., 105.}	623.667	0.776667	Reff	1.54029	1.75033	{1.12756, 2.4542}	28.5858	ar	695.	665.34	{69., 928.}	31.9885
34	R0	{1.50675, 19.8823}	{1.64986, 13.5196}	{{1.10369, 6.05643}, {2.4534, 19.255}}	{27.0389, 32.0134}	inf	62.	64.0019	{5.36417, 144.339}	76.7896	0.467288	newly	30.	30.4	{3., 63.}	68.5333	0.404643	Reff	1.22047	0.989237	{0.554047, 1.41937}	30.1158	ar	587.	577.72	{206., 864.}	34.3203
35	R0	{1.72889, 7.76068}	{1.97229, 12.2726}	{{1.18939, 5.62019}, {2.80569, 18.9406}}	{30.3699, 69.5586}	inf	6.	46.9126	{3.78379, 105.973}	701.064	0.800276	newly	7.	41.41	{3., 101.}	518.143	0.721097	Reff	1.70469	1.73387	{1.09441, 2.4542}	25.5643	ar	650.	653.25	{69., 928.}	37.0446
36	R0	{1.62644, 4.12303}	{1.94297, 11.4712}	{{1.27357, 5.3704}, {2.63959, 17.3398}}	{30.2496, 179.894}	inf	5.	38.4204	{2.59214, 90.7609}	684.71	0.769955	newly	11.	44.79	{2., 102.}	344.818	0.604624	Reff	1.61343	1.77518	{1.18566, 2.48668}	25.3798	ar	652.	658.55	{233., 902.}	31.8206
37	R0	{1.70878, 15.8072}	{1.72435, 15.0384}	{{1.26831, 7.36349}, {2.31477, 20.}}	{19.7762, 23.4741}	inf	53.	95.465	{30.3935, 190.554}	100.826	0.288265	newly	18.	43.16	{18., 78.}	145.889	0.34345	Reff	1.48835	1.12034	{0.749575, 1.60345}	28.9895	ar	674.	784.29	{541., 1019.}	24.5801
38	R0	{1.58967, 6.80568}	{1.91765, 13.201}	{{1.12882, 6.25107}, {2.79269, 19.0269}}	{35.1164, 100.265}	inf	6.	61.0612	{4.78513, 153.674}	930.661	0.823983	newly	10.	38.97	{3., 89.}	319.9	0.580917	Reff	1.55947	1.60391	{1.02997, 2.23343}	26.8297	ar	610.	653.61	{137., 917.}	39.159
39	R0	{1.344, 8.19498}	{1.80479, 13.3444}	{{1.11228, 5.72868}, {2.77502, 19.0269}}	{42.4899, 74.5935}	inf	10.	72.7839	{3.79943, 165.044}	653.129	0.779977	newly	8.	36.44	{3., 80.}	380.25	0.692843	Reff	1.2889	1.27699	{0.764875, 1.96011}	31.2559	ar	551.	608.31	{219., 926.}	44.8512
40	R0	{1.61509, 7.90825}	{1.94918, 11.5058}	{{1.27357, 5.3704}, {2.63959, 17.3398}}	{30.7637, 59.1639}	inf	9.	38.5574	{2.59214, 90.7609}	355.159	0.617119	newly	10.	44.86	{2., 102.}	383.2	0.620266	Reff	1.59894	1.78035	{1.18566, 2.48668}	25.7231	ar	581.	660.1	{233., 902.}	39.9484
41	R0	{1.60017, 5.90605}	{1.81379, 13.4943}	{{1.10693, 6.74711}, {2.60082, 19.1609}}	{30.8461, 130.093}	inf	8.	60.0966	{3.4726, 155.006}	670.174	0.764937	newly	8.	33.92	{2., 80.}	349.5	0.641542	Reff	1.56817	1.49483	{0.931688, 2.17624}	25.9019	ar	661.	635.74	{207., 898.}	33.1014
42	R0	{1.64436, 15.1337}	{1.83848, 13.368}	{{1.07874, 6.8071}, {2.64373, 19.1994}}	{29.6935, 23.0636}	inf	21.	67.9643	{3.69964, 177.021}	257.346	0.555153	newly	9.	39.34	{2., 89.}	363.556	0.648047	Reff	1.5605	1.46995	{0.931688, 2.05037}	25.042	ar	640.	646.51	{171., 899.}	34.2328
43	R0	{1.48917, 11.917}	{1.65417, 14.2827}	{{1.23913, 7.03796}, {2.3587, 20.}}	{22.5326, 41.1335}	inf	31.	80.626	{23.2872, 182.959}	174.407	0.381158	newly	19.	36.9	{14., 71.}	105.579	0.276615	Reff	1.38791	1.1008	{0.703481, 1.40716}	25.3086	ar	551.	730.87	{476., 993.}	38.0236
44	R0	{1.65577, 7.89317}	{1.90836, 13.3236}	{{1.12882, 6.31117}, {2.79269, 19.0443}}	{32.3408, 78.0359}	inf	6.	55.9963	{4.78513, 139.11}	846.602	0.789647	newly	9.	35.87	{3., 88.}	327.889	0.57793	Reff	1.63093	1.61559	{1.04836, 2.23343}	25.8494	ar	576.	648.27	{137., 917.}	43.5851
45	R0	{1.6909, 12.3275}	{1.84668, 13.3513}	{{1.05743, 7.10524}, {2.73737, 17.5057}}	{30.0283, 27.7274}	inf	23.	90.7383	{7.16405, 212.929}	322.863	0.596768	newly	14.	47.07	{5., 97.}	262.643	0.562089	Reff	1.5793	1.24446	{0.7776, 1.74375}	29.5991	ar	675.	646.07	{214., 913.}	31.4622
46	R0	{1.62351, 16.0698}	{1.76035, 13.228}	{{1.11461, 5.72868}, {2.75598, 19.0269}}	{28.8089, 27.1925}	inf	17.	69.6806	{3.85453, 162.199}	341.936	0.642391	newly	10.	35.58	{3., 76.}	281.8	0.614615	Reff	1.55532	1.26892	{0.77803, 1.96011}	32.2707	ar	591.	596.11	{220., 918.}	38.022
47	R0	{1.52372, 4.38599}	{1.94297, 11.4712}	{{1.27357, 5.3704}, {2.63959, 17.3398}}	{35.0731, 163.539}	inf	3.	38.4204	{2.59214, 90.7609}	1192.67	0.942114	newly	3.	44.79	{2., 102.}	1409.67	0.937486	Reff	1.51458	1.77518	{1.18566, 2.48668}	28.5554	ar	618.	658.55	{233., 902.}	35.199
48	R0	{1.44306, 11.788}	{1.84181, 13.4786}	{{1.12977, 8.09734}, {2.73517, 18.9813}}	{37.3049, 30.191}	inf	30.	79.7985	{3.85453, 190.31}	206.27	0.560362	newly	21.	39.31	{4., 81.}	126.524	0.488033	Reff	1.32617	1.28483	{0.793859, 1.96354}	31.1472	ar	534.	637.01	{220., 915.}	46.9607
49	R0	{1.63624, 11.3893}	{1.92434, 13.4132}	{{1.12882, 6.55128}, {2.79269, 19.0443}}	{33.6365, 39.9891}	inf	10.	58.6921	{4.78513, 141.529}	511.14	0.672943	newly	9.	37.32	{3., 89.}	344.	0.591683	Reff	1.60188	1.62324	{1.04836, 2.23343}	26.1443	ar	643.	653.7	{137., 917.}	35.8911
50	R0	{1.46113, 11.1322}	{1.7948, 13.191}	{{1.11228, 6.55128}, {2.65407, 18.4042}}	{35.0214, 37.1028}	inf	12.	72.4275	{6.72467, 159.843}	518.307	0.668479	newly	6.	41.53	{5., 94.}	603.167	0.72075	Reff	1.4246	1.35967	{0.872175, 2.0885}	26.7816	ar	501.	616.01	{197., 903.}	49.5509
